# Structural and functional annotation of the *Populus* × *xiaohei* var. gansuensis (C. Wang & H.L. Yang) C. Shang chloroplast genome

**DOI:** 10.1080/23802359.2025.2602959

**Published:** 2025-12-15

**Authors:** Yu-Rong Ren, Yong-Hao Nan, Chu-Yan Nie, Yu-Jun Kong, Jie Zhao, Jian-Rong Wei

**Affiliations:** College of Life Sciences, Hebei University, Baoding, China

**Keywords:** Chloroplast genome, *Populus*, phylogeny analysis

## Abstract

*Populus* × *xiaohei* var. gansuensis (C. Wang & H.L. Yang) C. Shang, a dominant tree in western China with high ecological and economic value, has no previously reported chloroplast genome. Here, we first assembled its complete circular chloroplast genome (156,632 bp) via whole-genome sequencing, which contains 133 genes. Phylogenetic analysis places it in the Tacamahaca clade, sister to *P. microphylla* and *P. germanica*. This resource aids poplar conservation, genetic research, and enriches *Populus* genomic data.

## Introduction

The genus *Populus*, ecologically and economically significant, is widely distributed across temperate to cold temperate zones of the Northern Hemisphere, forming a crucial component of forest ecosystems. *Populus* plantations in northwestern China playing a critical role in stabilizing desert margins, improving microclimates, and protecting oasis agriculture. However, taxonomic uncertainty persists within the genus due to frequent interspecific hybridization and substantial intraspecific variation (Wang et al. 2022). *Populus* × *xiaohei* var. *gansuensis* (C. Wang & H.L. Yang) C. Shang, 1982 (hereafter EBP; Wang and Tung 1982), is native to northwestern China and holds significant ecological and economic importance. EBP is believed to be a natural hybrid between *P. simonii* Carr. and *P. nigra* var. *thevestina* (Dode) Bean. This cultivar exhibits stable drought tolerance due to long-term artificial selection (Wang and Tung 1982). This variety exhibits stabilized drought and cold tolerance traits resulting from long-term artificial selection (Wang and Tung 1982). Chloroplast genomes, with conserved structure and maternal inheritance, are vital for resolving plant phylogenetics and evolutionary history (Gitzendanner et al. 2018). This study sequenced, assembled, and annotated the EBP chloroplast genome to provide fundamental data for systematic and evolutionary research within *Populus*.

## Materials and methods

**Figure 1.**
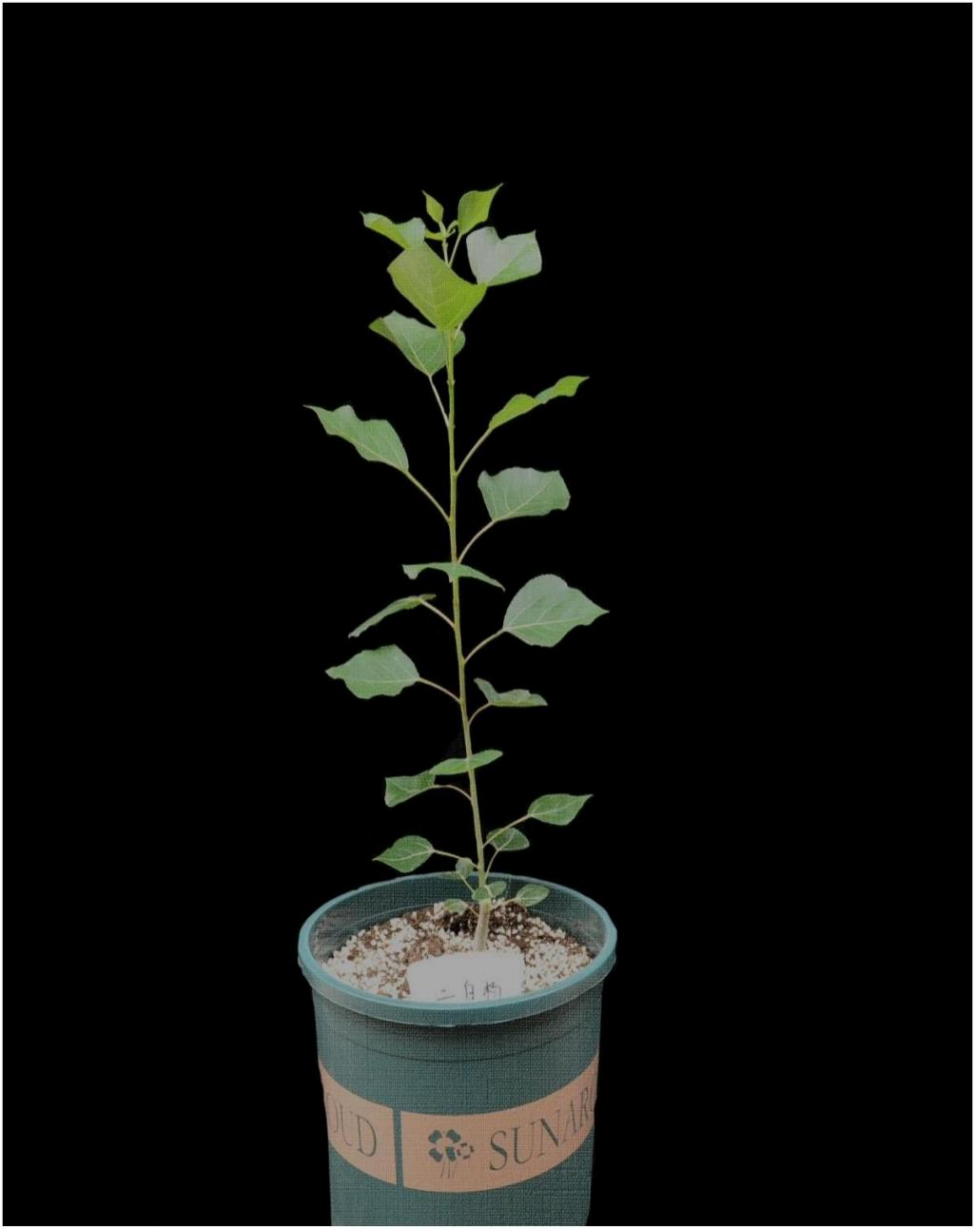
Pictures of *Populus* × *xiaohei* var. *gansuensis* (C. Wang & H.L. Yang) C. Shang. Leaves are ovate-elliptic with serrulate margins. The adaxial surface is smooth.

Shoot cuttings of EBP were provided by the Desert Forestry Experiment Center of the Chinese Academy of Forestry (106°35′E, 40°17′N), which were potted in the Plant-Insect Interaction Ecology Laboratory of Hebei University (Figure 1) and stored there (sample voucher number: HBSK402-EBY, contact person: Jian-Rong Wei, weijr@hbu.edu.cn).

Fresh leaf DNA was extracted using a commercial kit (CW2298S, Beijing, China). After extraction and verification, the samples were sequenced by Shanghai Applied Protein Technology Co., Ltd. (Shanghai, China) using the Illumina NovaSeq platform (Illumina, San Diego, CA), obtaining 21.58 GB of raw data. After quality screening, 21.08 GB of clean data were obtained. The chloroplast genome was assembled using the SPAdes software (Bankevich et al. 2012). Annotation and calibration utilized PGA (Qu et al. 2019) and CPStools (Huang et al. 2024), respectively, with the *Populus deltoides* genome (NC_040929; Zong et al. 2019) as a reference. Manual alignment and adjustment of the EBP genome were further performed using Geneious (Kearse et al. 2012) with the *Populus trichocarpa* (MW376841; Wang et al. 2022) as a reference to ensure accuracy and completeness. Before phylogenetic analysis, 18 required sequences were obtained from NCBI: 15 sequences from *Populus* species, two sequences from *Salix*, and one sequence from *Vitis* (as an outgroup). Multiple sequence alignments of chloroplast genomes were performed using the MAFFT online platform (https://mafft.cbrc.jp/alignment/server/) (Katoh and Standley 2013). Ambiguous regions in the alignment were trimmed using GBlocks (Castresana 2000), and conserved regions were trimmed using default parameters. In phylogenetic inference using IQ-TREE (Nguyen et al. 2015), ModelFinder automatically selected the best-fit model, which was identified as K3Pu + F + I + G4 based on the Bayesian information criterion (BIC). The number of bootstrap replicates for both the ultra-fast bootstrap and SH-aLRT tests was set to 1000, and node support was evaluated using a correlation coefficient threshold of 0.99. The generated data were visualized in FigTree to generate a phylogenetic tree. Genomic features, including tetrad junction sites (Li et al. 2023), nucleotide diversity, and relative synonymous codon usage (RSCU), were analyzed using Genepioneer’s bioinformatics platform (Nanjing, China). transfer RNA (tRNA) secondary structures were predicted using tRNAscan-SE (http://lowelab.ucsc.edu/tRNAscan-SE) and visualized via VARNA (Darty et al. 2009). Comparative genomics, including alignments of EBP, *P. trichocarpa* (NC_040866), *P. deltoides* (MN417118), and *P. alba* (NC_008235), were conducted using VISTA tools (https://genome.lbl.gov/vista/index.shtml) (Zhang et al. 2024). Coverage depth maps were generated using SAMtools (Li et al. 2009). A circular chloroplast genome map and cis-splicing and trans-splicing gene maps were constructed using CGView tool (Liu et al. 2023).

## Results

**Figure 2.**
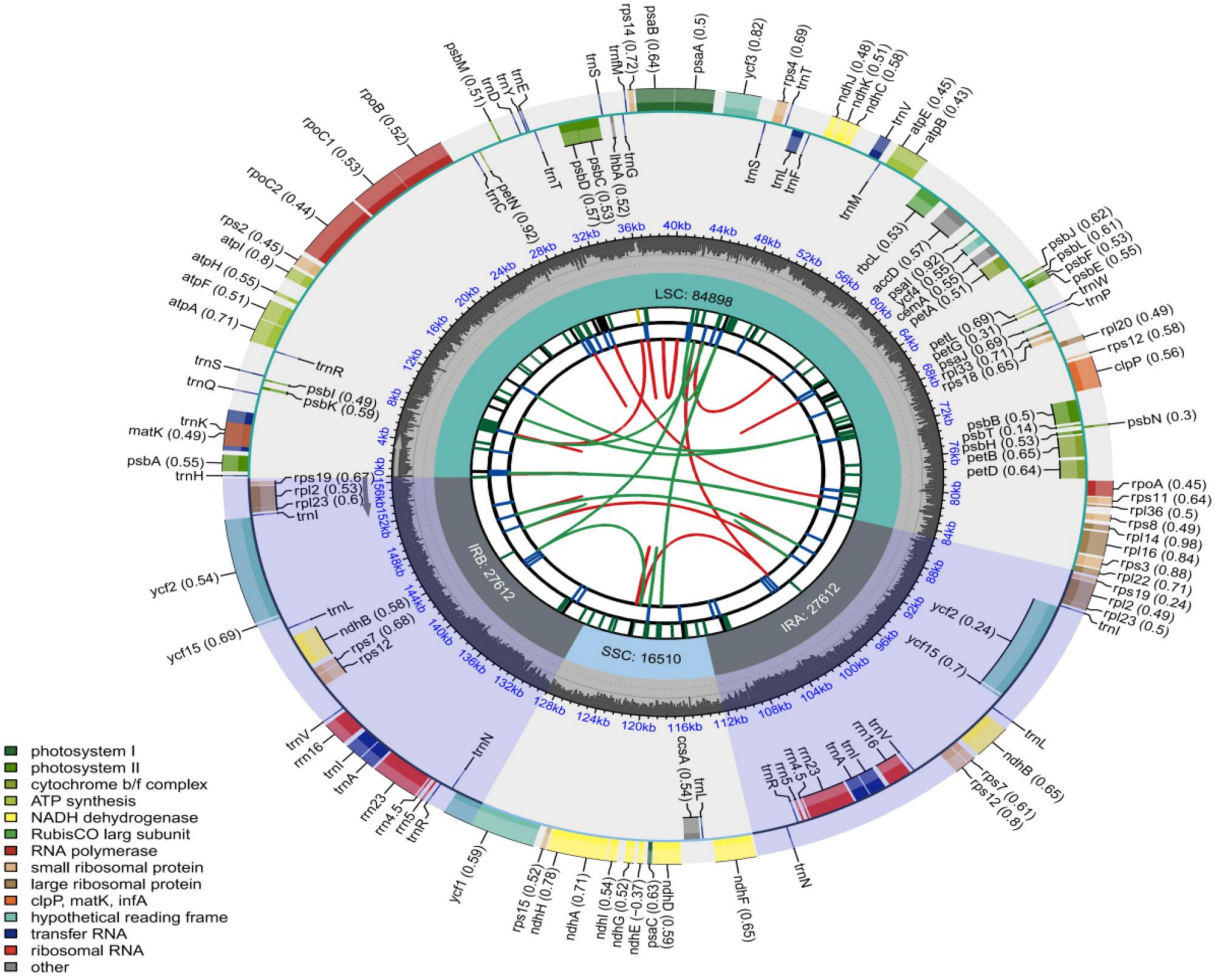
Circular chloroplast genome map of *Populus* × *xiaohei* var. *gansuensis* (C. Wang & H.L. Yang) C. Shang. Different functional genes are identified by different colors, and their functions are displayed in the lower left corner. The innermost circle depicts dispersed repeats, including forward repeats (connected by red arcs) and palindrome repeats (connected by green arcs). The second circle explains the long tandem repeats represented by blue short bars. In the third circle, simple sequence repeats are represented by short colored bars, each bar indicating a different repeat unit size (RUS). The fourth circle depicts regions including small single-copies (SSC), inverted repeat sequences (IRa and IRb), and large single-copies (LSC). The fifth track plots the GC content of the entire plastid. In the sixth circle, the plastid genes are annotated, and the parentheses after each gene name indicate the optional codon usage bias. Genes are color-coded according to their functions, as shown in the legend. The genes in the inner and outer circles are transcribed clockwise and counterclockwise, respectively.

The complete chloroplast genome sequence of EBP was 156,632 bp in length. It is a typical tetrad structure. The GC content of the species was 37.98%, consisting of a large single-copy (LSC) region (84,898 bp), a small single-copy (SSC) region (16,510 bp), and a pair of inverted repeats (IRs) (each 27,612 bp) (Figure 2). The average and minimum read mapping depths of the assembled genome were 9545× and 30×, respectively (Figure S1).

The EBP genome encodes a total of 134 genes, comprising 88 protein-coding genes, 38 tRNA genes, and eight ribosomal RNA (rRNA) genes. By analyzing the chloroplast genomes of four species (EBP, *P. trichocarpa*, *P. deltoides*, and *P. alba var. pyramidalis*) of poplar trees, it was found that there were certain differences in the genomic structure among these four species, for instance, the positions of genes such as *rps19*, *rpl22*, *trnN*, *ndhF*, *ycf1* were different (Figure S2). Nucleotide diversity (Pi) analysis identified highly variable regions as key evolutionary markers. Within the LSC, multiple Pi peaks are observed, such as in the *psbZ-trnG*, *trnC*, and *ndhC-trnV-exon2* regions, indicating high nucleotide diversity and likely hypervariable regions within the genome (Figure S3).

The secondary structure of tRNA genes is mainly a typical cloverleaf structure, and as shown in the figure, trnL1, *trnL2*, *trnG*, *trnS2*, *trnY*, etc., also have extra loops (Figure S4). At the genetic level, certain genes, such as *rps2*, *rps14*, *rps4*, and *rps18*, display high conservation across all four poplar species. This suggests that these genes are relatively stable during evolution and play a crucial role in the survival and growth of poplar trees (Figure S5). RSCU analysis reveals codon usage bias (e.g. *Ala*, *Arg*), providing insights for gene expression regulation studies (Figure S6). Ten genes involving cis-splicing (*atpF*, *rpoC1*, *ycf3*, *clpP*, *petB*, *petD*, *rpl16*, *rpl2*, *ndhB*, *ndhA*) and one gene involving trans-splicing (*rps12*) were identified (Figures S7 and S8).

**Figure 3.**
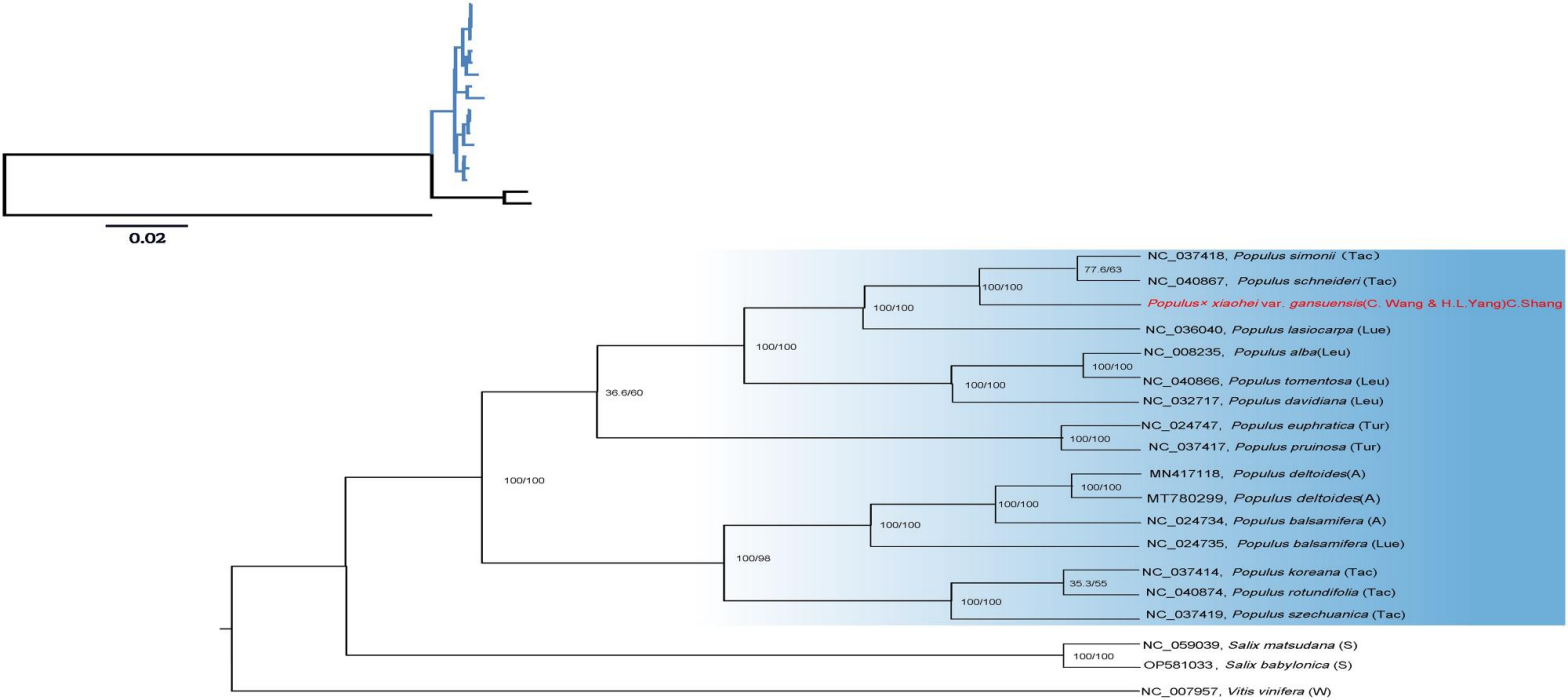
Phylogenetic analysis of *Populus gansuensis* and other related species based on complete chloroplast genome sequences. The bootstrap values are shown on the nodes. Node numbers represent P: section Leuce; A: section Aigeiros; Tac: section Tacamahaca; L: section Leucoides; Tur: section Turanga; S: Salix; W: outgroup. Reference information can be found in Supplementary Table 2. The genome of the species identified with red-bold font is presented in this study. The following sequences were used in this study: *P. simonii* NC_037418, *P. schneideri* NC_040867 (Zong et al. 2019), *P. lasiocarpa* NC_036040, *P. alba* NC_008235 (Okumura et al. 2006), *P. tomentosa* NC_040866 (Zong et al. 2019), *P. davidiana* NC_032717 (Zong et al. 2019), *P. euphratica* NC_024747 (Zhang and Gao 2016), *P. pruinosa* NC_037417, *P. deltoides* MN417118 (Su et al. 2019), *P. deltoides* MT780299, *P. fremontii* NC_024734 (Huang et al. 2014), *P. balsamifera* NC_024735 (Huang et al. 2014), *P. koreana* NC_037414, *P. cathayana* NC_040874 (Zong et al. 2019), *P. szechuanica* NC_037419, *Salix matsudana* NC_059039, *Salix babylonica* OP581033, and *Vitis vinifera* NC_007957 (Jansen et al. 2006) (outgroup).

Phylogenetic analysis based on the complete chloroplast genome dataset revealed that the clade encompassing EBP acquired a 100% bootstrap support value; this branch exhibited a distinct sister-group relationship with *Populus simonii* (NC_037418.1) and *Populus schneideri* (NC_040867.1) (Figure 3).

## Discussion and conclusions

*Populus* species are predominantly distributed across the temperate Northern Hemisphere, with occasional occurrences in South America and South Africa (Ding 1995). Valued for their rapid growth and strong adaptability, they play critical roles in ecological stability and economic development. Plant chloroplast genomes, characterized by high conservation, offer robust tools for investigating interspecific relationships, taxonomy, and evolutionary genetics (Song et al. 2017). In this study, the chloroplast genome of EBP was sequenced and assembled, revealing structural features consistent with most *Populus* species (Okumura et al. 2006; Tuskan et al. 2006; Zong et al. 2019). Key differences were observed in genome size (EBP: 156,632 bp, the largest; *P. alba* var. *pyramidalis*: 156,342 bp, the smallest), GC content, repeat sequences, and IR boundaries (*P. trichocarpa* exhibits the largest IR region, while EBP has the smallest). This variation is likely driven by IR region shrinkage, a major factor influencing chloroplast genome size (Wu et al. 2018). EBP retains the conserved four-part chloroplast structure (LSC, SSC, and two IRs) typical of *Populus*, confirming structural conservation within the genus. These differences may reflect adaptive evolution to arid habitats in western China, providing clues for elucidating interspecific differentiation mechanisms. Further analysis identified distinct nucleotide diversity (Pi) peaks in LSC (e.g. *psbZ-trnG*, *trnG*) and SSC (e.g. *psaC-ndhD*, *rps15-ycf1*) regions, indicating high genetic variation in these loci – potential key regions for adaptive evolution and species divergence. These findings enhance our understanding of Populus genetic resources and provide foundational data for genus classification.

## Supplementary Material

Supplementary Material clean.doc

## Data Availability

The chloroplast genome of EBP in this study is publicly available in NCBI (https://www.ncbi.nlm.nih.gov/) under accession number PV200281. The accession no. of BioProject, Bio-Sample, and SRA are PRJNA1238499, SAMN47474361, and SRR32784498, respectively.
