# Supplementary material for: Structural and functional annotation of the Populus × xiaohei var. gansuensis (C. Wang & H.L. Yang) C. Shang chloroplast genome
Source: Mitochondrial DNA B Resour. 2025 Dec 15;11(1):64–8. doi: 10.1080/23802359.2025.2602959 (PMC12707073; doi:10.1080/23802359.2025.2602959)
Supplement: Supplementary Material clean.doc [file TMDN_A_2602959_SM3941.doc]

**Supplementary Table1.** List of gene in chloroplast genome of *P. × xiaohei* var. *gansuensis* (C. Wang&H. L. Yang) C. Shang

| **Gene Functions** | **Group of genes** | **Name of genes** |
| --- | --- | --- |
| Photosynthesis | Subunits of ATP synthase | *atpA, atpB, atpE, atpF*, atpH, atpI* |
|  | Subunits of NADH dehydrogenase | *ndhA *, ndhB**(×2)*, ndhC, ndhD, ndhE, ndhF, ndhG, ndhH, ndhI, ndhJ,*  *ndhK*(×2) |
|  | Subunits of cytochrome | *petA, petB***, petD**(×2)*, petG, petL, petN* |
|  | Subunits of photosystem I | *psaA, psaB, psaC, psaI, psaJ* |
|  | Subunits of photosystem II | *psbA, psbB, psbC*(×2)*, psbD, psbE, psbF, psbH, psbI, psbJ ,psbK*(×2)*, psbL,*  *psbM, psbT, psbZ* |
|  | Subunit of rubisco | *rbcL* |
| Self-replication | Large subunit of ribosome | *rpl14, rpl16***, rpl20 ,rpl2**(×2) *,rpl22, rpl23*(×2)*, rpl33, rpl36* |
|  | Small subunit of ribosome | *rps11, rps12***(×2)*, rps14, rps15, rps18, rps19*(×2)*, rps2, rps3, rps4,*  *rps7*(×2)*, rps8* |
|  | DNA dependent RNA polymerase | *rpoA, rpoB, rpoC1**(×2)*, rpoC2* |
|  | Ribosomal RNAs | *rrn4.5*(×2)*, rrn5*(×2) *,rrn16*(×2)*, rrn23**(×2) |
|  | Transfer RNAs | *trnA-UGC**(×2)*, trnC-GCA, trnD-GUC, trnE-UUC, trnF-GAA,*  *trnG-GCC, trnG-UCC***,trnH, trnH-GUG, trnI-CAU*(×2)*,trnI-GAU**(×2)*, trnK-UUU***, trnL-CAA*(×2)*, trnL-UAA***, trnL-UAG, trnM-CAU,*  *trnN-GUU*(×2)*, trnP-UGG, trnQ-UUG, trnR-ACG*(×2)*, trnR-UCU,*  *trnS-GCU, trnS-GGA, trnS-UGA, trnT-GGU, trnT-UGU, trnV-GAC*(×2)*,*  *trnV-UAC***, trnW-CCA, trnfM-CAU, trny-GUA* |
| Other genes | Maturase | *matK* |
|  | Protease | *clpP1 *** |
|  | Envelop membrane protein | *cemA* |
|  | Subunit of Acetyl-CoA-carboxylase | *accD* |
|  | c-type cytochrome synthesis gene | *ccsA* |
|  | Translational initiation | *infA* |
| Unknown function | Conserved open reading frames | *ycf1, ycf2, pbf1, pafI****, pafII* |

Note: *gene with a single intron; **gene with two introns; (×2) duplicated gene.

**Supplementary Table 2 . Phylogenetitree information**

| **Species** | **Genbank** | **Reference** | **DOI** |
| --- | --- | --- | --- |
| *Populus simonii* isolate | NC_037418.1 |  |  |
| *Populus schneideri* | NC_040867.1 | Zong et al., 2019 | 10.3389/fpls.2019.00005 |
| *Populus lasiocarpa* | NC_036040.1 |  |  |
| *Populus alba* | NC_008235.1 | Okumura et al., 2006 | 10. 1007/s11248-006-9009-3. |
| *Populus tomentosa* Carrière | NC_040866.1 | Zong et al., 2019 | 10.3389/fpls.2019.00005. |
| *Populus davidiana* Dode | NC_032717.1 | Zong et al., 2019 | 10.3389/fpls.2019.00005. |
| *Populus euphratica* Olivier | NC_024747.1 | Zhang et al., 2016 | 10.3109/19401736.2014.913159. |
| *Populus pruinosa* isolate | NC_037417.1 |  |  |
| *Populus deltoides* cultivar | MN417118.1 | Su et al., 2019 | 10.1080/23802359.2019.1700840. |
| *Populus deltoides* clone I69 | MT780299.1 |  |  |
| *Populus fremontii* | NC_024734.1 | Huang et al., 2014 | 10.1111/nph.12956 |
| *Populus balsamifera* | NC_024735.1 | Huang et al., 2014 | 10.1111/nph.12956 |
| *Populus koreana* Rehd. | NC_037414.1 |  |  |

| *Populus cathayana* | NC_040874.1 | Zong et al., 2019 | 10.3389/fpls.2019.00005. |
| --- | --- | --- | --- |
| *Populus szechuanica* | NC_037419.1 |  |  |
| *Salix matsudana* Koidz | NC_059039.1 |  |  |
| *Salix babylonica* | OP581033.1 |  |  |
| *Vitis vinifera* | NC_007957.1 | Jansen et al., 2019 | 10. 1186/1471-2148-6-32. |

**References**

Huang DI, Hefer CA, Kolosova N, Douglas CJ, Cronk QCB. 2014. Whole plastome sequencing reveals deep plastid divergence and cytonuclear discordance between closely related balsam poplars, *Populus balsamifera* and *P. trichocarpa* (*Salicaceae*). New Phytol. 204(3):693-703. doi: [10.1111/nph.12956](https://doi.org/10.1111/nph.12956).

Jansen RK, Kaittanis C, Saski C, Lee SB, Tomkins J, Alverson AJ, Daniell H. 2006. Phylogenetic analyses of *Vitis* (*Vitaceae*) based on complete chloroplast genome sequences: effects of taxon sampling and phylogenetic methods on resolving relationships among rosids. BMC Evol Biol. 6:32. doi: [10.1186/1471-2148-6-32](https://doi.org/10.1186/1471-2148-6-32).

Okumura S, Sawada M, Park YW, Hayashi T, Shimamura M, Takase H, Tomizawa K. 2006. Transformation of poplar (*Populus alba*) plastids and expression of foreign proteins in tree chloroplasts. Transgenic Res. 15(5):637-46. doi: [10.1007/s11248-006-9009-3](https://doi.org/10.1007/s11248-006-9009-3).

Su T, Han M, Min J, Cao D, Pan H, Liu Y. 2019. The complete chloroplast genome sequence of *Populus deltoides* 'Siyang-2'. Mitochondrial DNA B Resour. 5(1):283-285. doi:

[10.1080/23802359.2019.1700840](https://doi.org/10.1080/23802359.2019.1700840).

Zhang QJ, Gao LZ. 2016. The complete chloroplast genome sequence of desert poplar (*Populus euphratica*). Mitochondrial DNA A DNA Mapp Seq Anal. 721-723. doi:

[10.3109/19401736.2014.913159](https://doi.org/10.3109/19401736.2014.913159).

Zong D, Gan P, Zhou A, Zhang Y, Zou X, Duan A, Song Y, He C. 2019. Plastome Sequences Help to Resolve Deep-Level Relationships of *Populus* in the Family *Salicaceae*. Front Plant Sci. 10:5. doi: [10.3389/fpls.2019.00005](https://doi.org/10.3389/fpls.2019.00005).


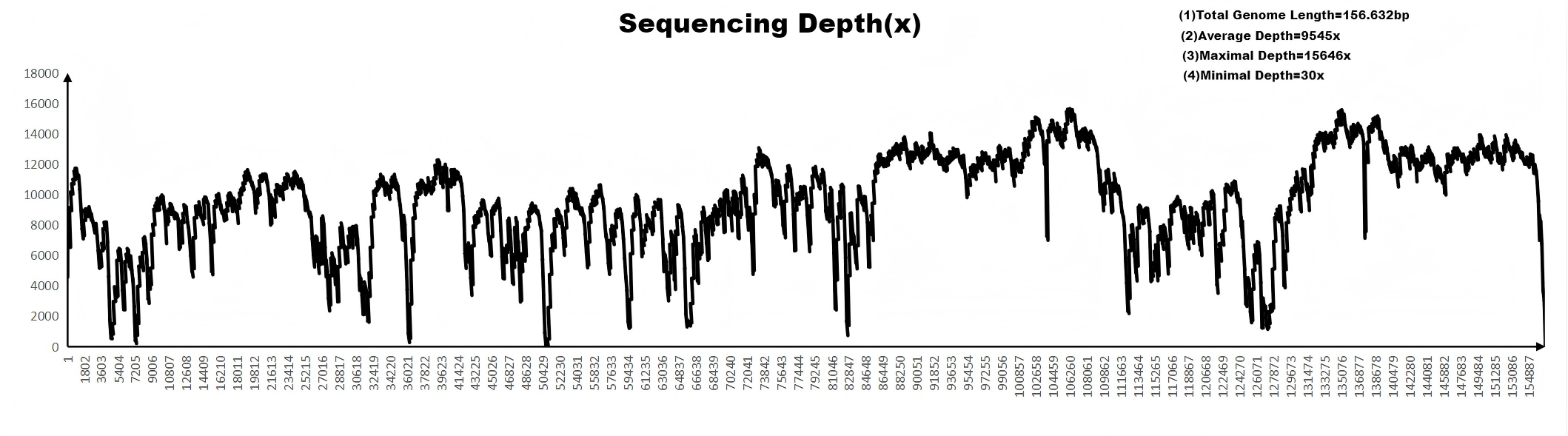
**Supplementary Figure 1.** The chloroplast genome coverage depth map of *P.* × *xiaohei* var. *gansuensis*(C. Wang&H. L. Yang) C. Shang. The sequencing depth profile verified the reliability of genome coverage. The maximum sequencing depth in this study was 15646×, the minimum sequencing depth was 30×, and the average sequencing depth was 9545×.


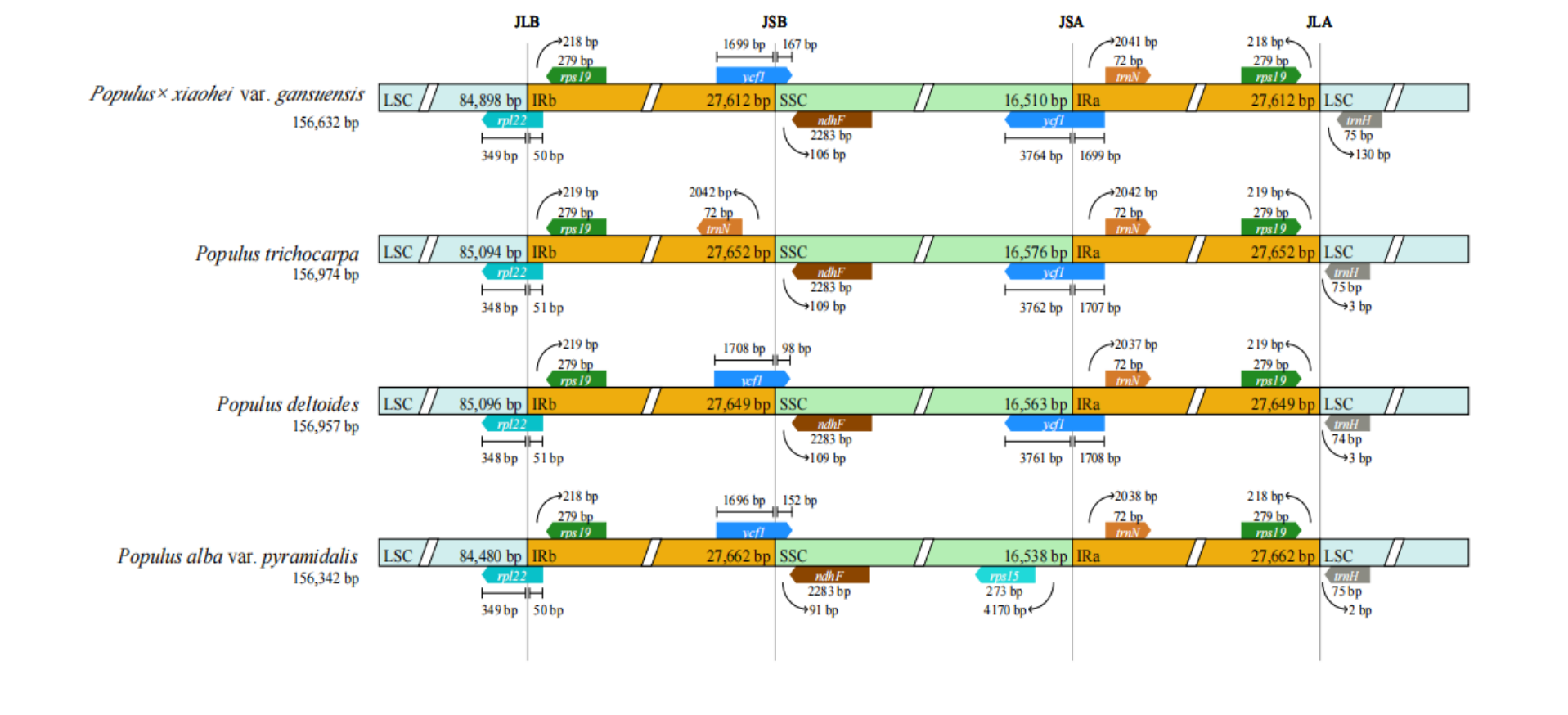


**Supplementary Figure 2.** Chloroplast Genome Structure and Size in *Populus.* Junction regions and size variation Conserved junction regions (JLB, JSB, JSA, JLA; 72–2,041 bp) and genome sizes (156.3–157.0 kb) across four *Populu*s species.


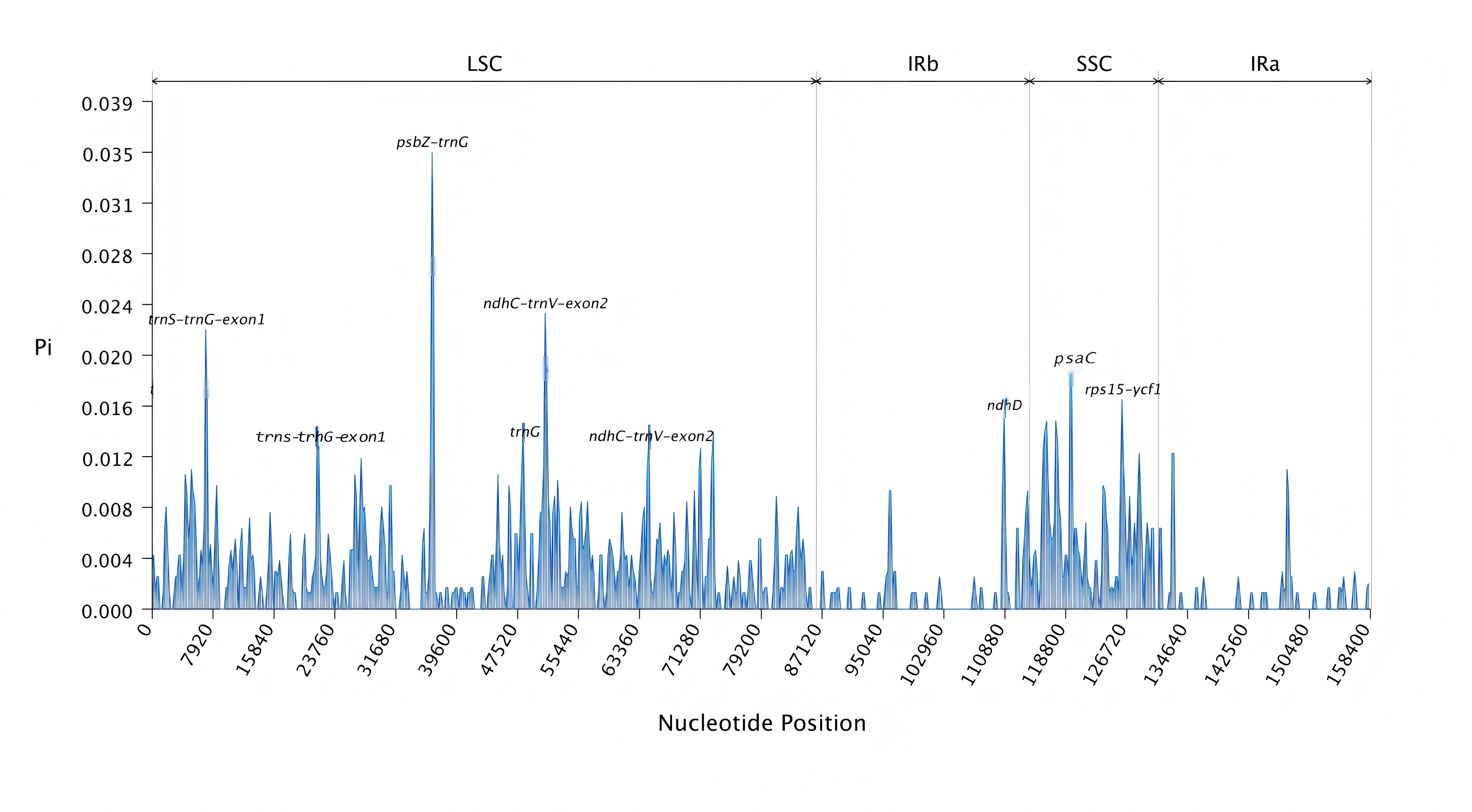
**Supplementary Figure 3.** Nucleotide Polymorphism (Pi) values were calculated by comparing *P.* × *xiaohei* var. *gansuensis* (C. Wang&H. L. Yang) C. Shang with *Populus trichocarpa* (GenBank: MW376841), *Populus deltoides* (GenBank: NC_040929) and *Populus alba* var. *Pyramidalis* (GenBank: MG262344).





**Supplementary Figure 4.** Visualization of tRNA secondary structure of *P. × xiaohei* var. *gansuensis* (C. Wang & H. L. Yang) C. Shang.


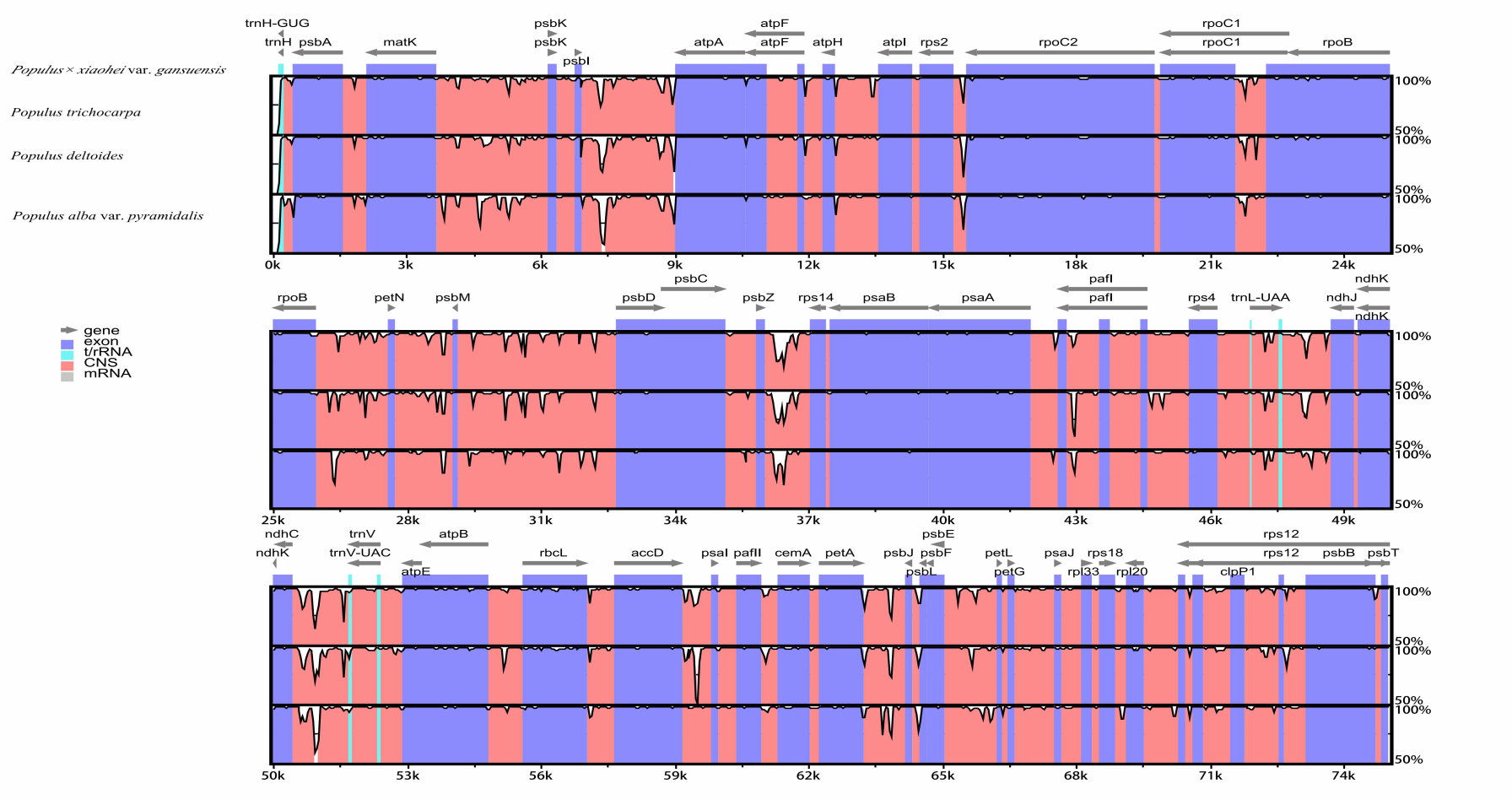


**Supplementary Figure 5.** Comparative Sequence Alignment of Chloroplast Genes in *Populus* Species.This sequence alignment map off our *Populus* species (*P. × xiaohei* var. *gansuensis* (C. Wang & H. L. Yang) C. Shang, *P. trichocarpa* (GenBank: MW376841), *P. deltoides* (GenBank: NC_040929) and *P. alba* (GenBank: MG262344)) shows chloroplast genes, with red/blue regions representing conserved/variable regions and annotated genes.


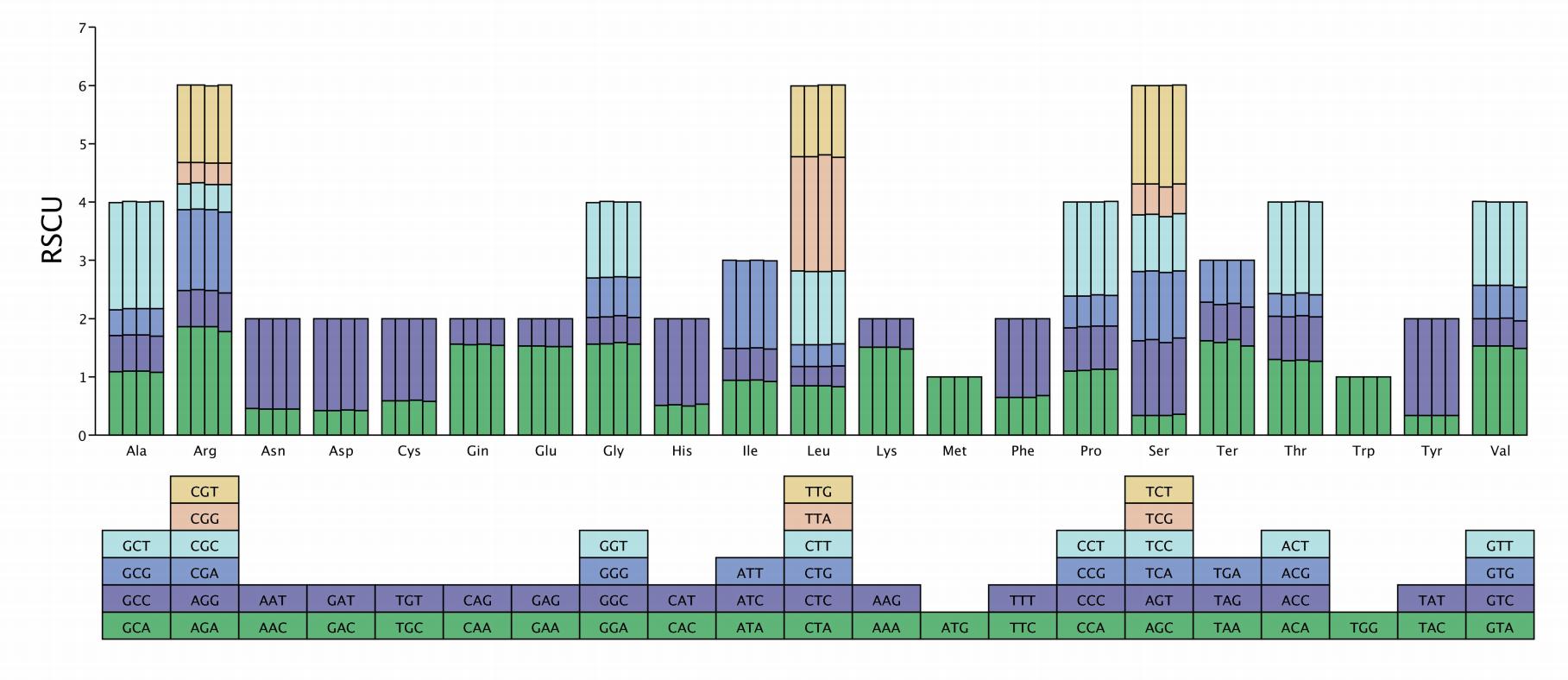


**Supplementary Figure 6.** Relative Synonymous Codon Usage (RSCU). This barchart shows the RSCU for different codons in *P.* × *xiaohei* var. *gansuensis* (C. Wang & H. L. Yang) C. Shang, obtained by comparing it with *Populus trichocarpa* (GenBank: MW376841), *Populus deltoides* (GenBank: NC_040929) and *Populus alba* var. *pyramidalis* (GenBank: MG262344). Codons are color - coded, with the x - axis listing amino acids and their codons, and the y - axis indicating RSCU values. Different colors represent different codons.


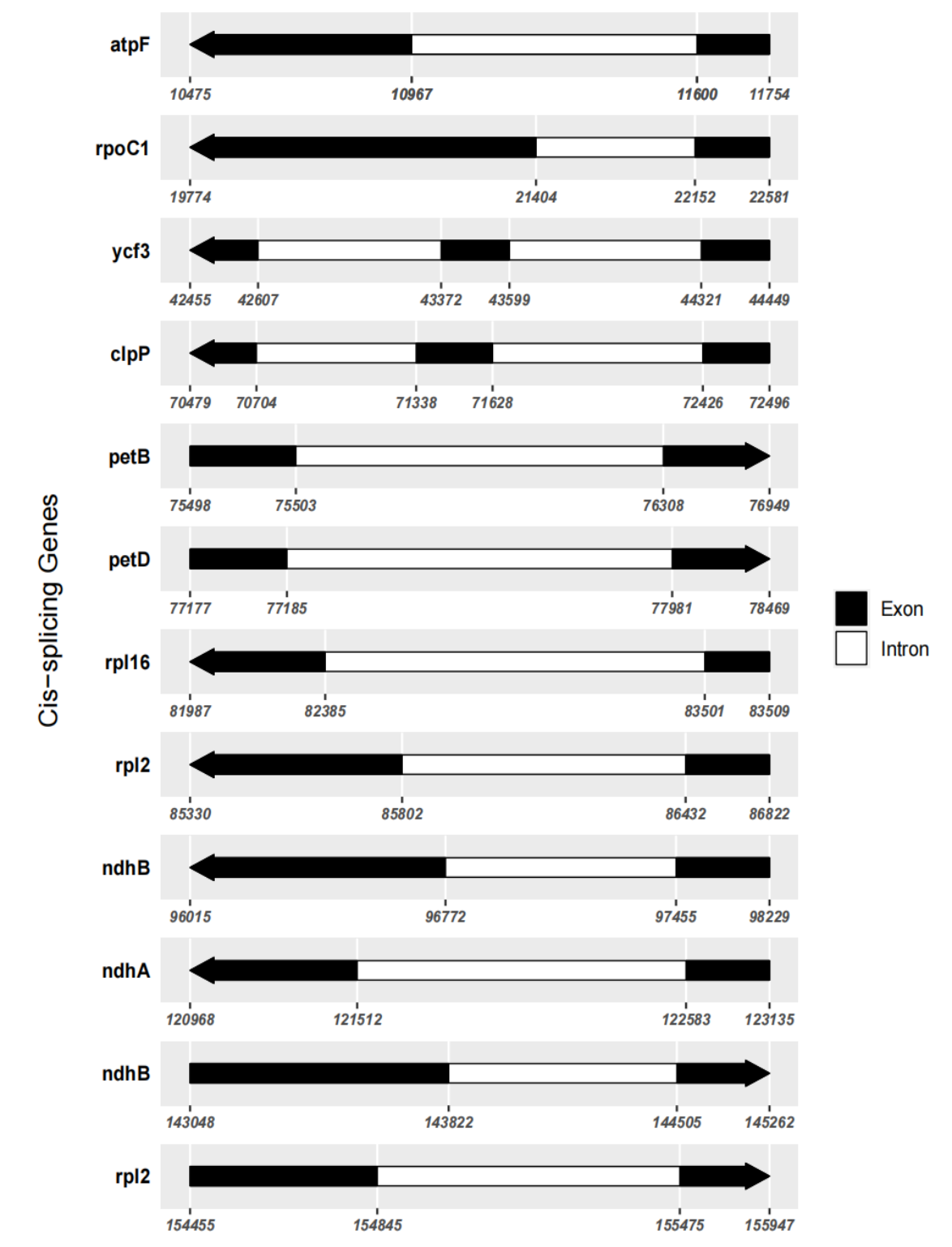


**Supplementary Figure 7.** Depicts a schematic illustration of the cis-splicing genes within the chloroplast genome. Here, exons are represented in black, while introns are shown in white. An arrow is included to denote the sense orientation of the gene. Note that the exon and intron lengths are not drawn to scale.


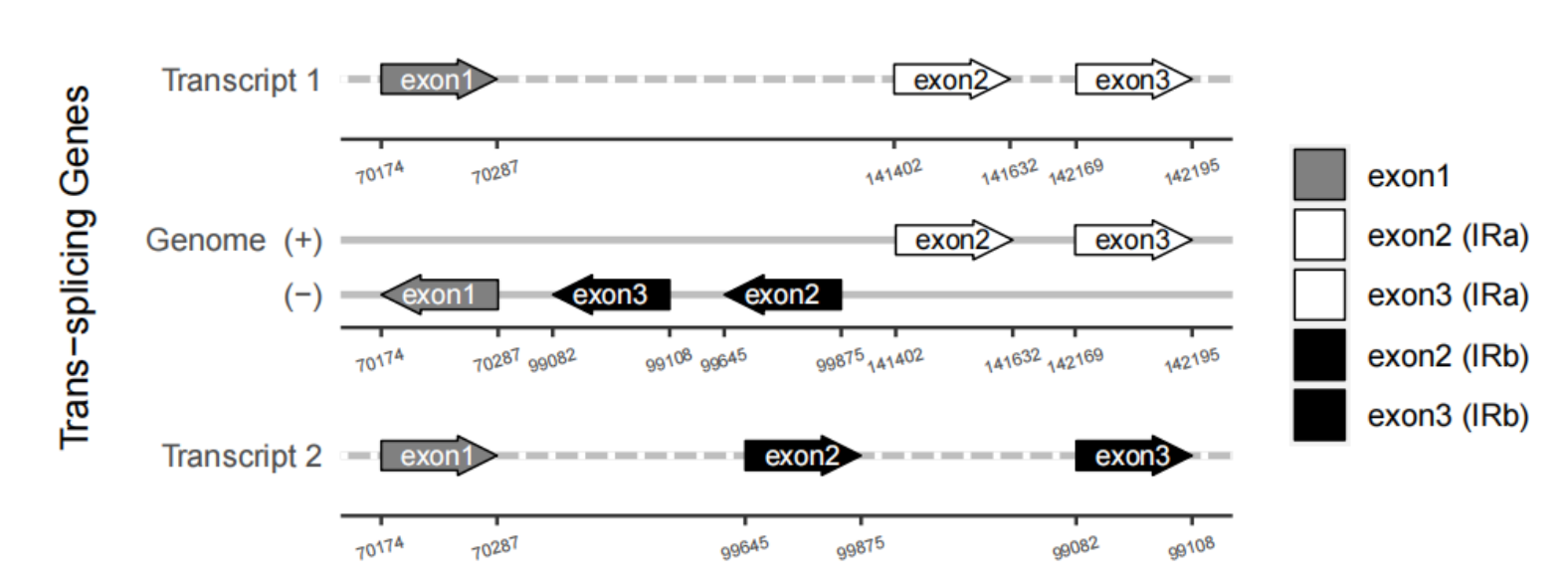


**Supplementary Figure 8.** Trans-splicing gene map of (*rps12)* in the chloroplast genome of *P. × xiaohei* var. *gansuensis* (C. Wang & H.L.Yang) C. Shang.
